# TMS: Ensemble Deep Learning Model for Accurate Classification of Monkeypox Lesions Based on Transformer Models with SVM

**DOI:** 10.3390/diagnostics14232638

**Published:** 2024-11-23

**Authors:** Elsaid Md. Abdelrahim, Hasan Hashim, El-Sayed Atlam, Radwa Ahmed Osman, Ibrahim Gad

**Affiliations:** 1 Computer Science Department, Science College, Northern Border University (NBU), Arar 73213, Saudi Arabia; elsaid.abdelrahim@nbu.edu.sa; 2 Computer Science Department, Faculty of Science, Tanta University, Tanta 31527, Egypt; satlam@yahoo.com; 3 Department of Computer Science, College of Computer Science and Engineering, Taibah University, Yanbu 46421, Saudi Arabia; hhashim@taibahu.edu.sa; 4 Basic and Applied Science Institute, College of Engineering and Technology, Arab Academy for Science and Technology (AAST), Alexandria 1029, Egypt; radwa.ahmed@aast.edu

**Keywords:** monkeypox, machine learning, prediction, imbalanced data, deep learning, optimization

## Abstract

Background/Objectives:The emergence of monkeypox outside its endemic region in Africa has raised significant concerns within the public health community due to its rapid global dissemination. Early clinical differentiation of monkeypox from similar diseases, such as chickenpox and measles, presents a challenge. The Monkeypox Skin Lesion Dataset (MSLD) used in this study comprises monkeypox skin lesions, which were collected primarily from publicly accessible sources. The dataset contains 770 original images captured from 162 unique patients. The MSLD includes four distinct class labels: monkeypox, measles, chickenpox, and normal. Methods: This paper presents an ensemble model for classifying the monkeypox dataset, which includes transformer models and support vector machine (SVM). The model development process begins with an evaluation of seven convolutional neural network (CNN) architectures. The proposed model is developed by selecting the top four models based on evaluation metrics for performance. The top four CNN architectures, namely EfficientNetB0, ResNet50, MobileNet, and Xception, are used for feature extraction. The high-dimensional feature vectors extracted from each network are then concatenated and optimized before being inputted into the SVM classifier. Results: The proposed ensemble model, in conjunction with the SVM classifier, achieves an accuracy of 95.45b%. Furthermore, the model demonstrates high precision (95.51%), recall (95.45%), and F1 score (95.46%), indicating its effectiveness in identifying monkeypox lesions. Conclusions: The results of the study show that the proposed hybrid framework achieves robust diagnostic performance in monkeypox detection, offering potential utility for enhanced disease monitoring and outbreak management. The model’s high diagnostic accuracy and computational efficiency indicate that it can be used as an additional tool for clinical decision support.

## 1. Introduction

The world is still trying to recover from the COVID-19 pandemic, but now it has to deal with a new virus called monkeypox [1,2]. Monkeys and rodents are the main sources of virus transmission; however, it was noted that once it infects humans, it is easily transmitted from human to human, causing a very rapid spread of the virus [3]. The initial human monkeypox case documented in the Democratic Republic of the Congo in 1970 [4], and the disease was initially detected in Copenhagen, Denmark [5]. It is known that the people most vulnerable to infection are those living near tropical rainforests.

Various modes of transmission contribute to the spread of the virus, including direct physical contact with an infected animal or human. Moreover, the infection occurs due to the transmission of saliva, nasal secretions, or respiratory droplets [4,6]. Additionally, animal bites can cause infection and transmission of the virus. The infected person may experience different short-term symptoms, such as back pain, muscle aches, fever, weariness, low energy, headache, swollen lymph nodes, and, in the long run, red bumps on the skin [7].

Information gathered from Africa, Europe, and the United States identified people infected by monkeypox in 2022. However, there are no specialized or licensed treatments for monkeypox [8], which causes concern among people around the world. Nevertheless, human immunity can successfully identify the monkeypox virus. Additionally, in some countries, monkeypox treatment typically involves the use of smallpox vaccinations [9].

Machine learning, often referred to as ML, is considered a promising solution for detecting diseases and establishing the required treatments. Researchers have concentrated on machine learning, which use historical data to address real-world problems and provides accurate predictions regarding the number of pandemic cases, allowing them to track the virus’s spread and incubation. In recent years, deep learning and ensemble learning have gained significant attention for various classification challenges in medical imaging, particularly due to advancements in computational power and improved algorithms. The CNN model and other deep learning models have demonstrated remarkable efficacy in image classification and disease detection tasks, such as the prediction of COVID-19 cases [10,11,12,13], breast cancer [14], brain tumors [15], Alzheimer’s progression [16,17], and various other diseases [18,19,20,21].

Recent years have seen numerous studies focusing on the investigation of the monkeypox virus. However, these studies have lacked the precise measurements required for virus detection. When it comes to monkeypox data, efficient and accurate systems are needed for virus detection. Therefore, several frameworks have been implemented to prevent the spread of various diseases [22,23]. Furthermore, AI has the potential to serve as a powerful tool for combating monkeypox infections and as an efficient method for virus detection. In the future, it may also be useful for improving the clinical care of patients [24].

This study proposes a novel ensemble model combining transformer models with a support vector machine (SVM) for highly accurate monkeypox lesion classification. The proposed model utilizes the advantages of several CNN architectures for feature extraction and optimizes these features using an SVM classifier, in contrast to previous research that primarily relies on traditional CNN or single deep learning architectures. This combination addresses the problem of imbalanced datasets, a crucial issue in medical image analysis, while also improving the model’s classification accuracy. Furthermore, the use of transformer models in the ensemble adds a layer of robustness, allowing the model to capture more complex patterns that may be missed by traditional CNN-based approaches. Consequently, the contributions of this study are as follows:

- Developing an ensemble model for the classification of monkeypox images.
- Providing a systematic evaluation of the proposed framework’s performance through several experimental results on the open-access Monkeypox dataset.
- Demonstrating that the proposed framework achieves satisfactory performance, outperforming state-of-the-art models in terms of evaluation metrics.

The remaining sections of this work are organized as follows: Section 2 discusses related work relevant to the paper’s subject. Section 3 describes the dataset and its processing during the experiment. Section 4 provides a description of the proposed framework. The analysis and discussion of the results are detailed in Section 5. Finally, Section 7 summarizes the conclusions of this study as well as possible future works and recommendations.

## 2. Related Work

Deep learning (DL) has been growing in popularity across various fields, offering powerful solutions to challenging real-world problems, particularly in areas requiring high precision and accuracy, such as healthcare, civil engineering, and industrial diagnostics. Numerous models and systems have been developed using ML and DL to predict different diseases. Hadeer et al. [25] developed an end-to-end framework for medical image classification and early detection of Alzheimer’s disease using a CNN model. Additionally, Tamer et al. [26] presented and evaluated two hybrid deep learning methods for Alzheimer’s progression, culminating in an interpretable clinical decision support system based on predicted scores. Furthermore, metaheuristics and deep learning have been employed for early detection of brain tumors due to their inherent complexity [27]. DL models have also been utilized for early detection of breast cancer. Ahmad et al. [28] applied ML, DL, and deep reinforcement learning for the classification and detection of breast cancer.

Eid et al. [29] proposed an improved Long Short-Term Memory (LSTM) model for accurate prediction of monkeypox cases. Additionally, a novel binary hybrid algorithm was proposed by Abdelhamid et al. [30], which utilizes meta-heuristic optimization algorithms for feature selection, significantly improving the classification accuracy of monkeypox images. The project presented in [31] aimed to build the Monkeypox Skin Image Dataset 2022, addressing the scarcity of monkeypox skin image data that creates a bottleneck in using machine learning for monkeypox detection. Moreover, to enable accurate diagnoses and treatment, Alakus et al. [32] analyzed DNA sequences of MPV (causing monkeypox) and HPV (causing warts) and performed their classification using deep learning.

To forecast the transmission of monkeypox, Dada et al. [33] proposed a stacking ensemble learning framework combining machine learning techniques. Sitaula et al. [34] aimed to detect the monkeypox virus using 13 different pre-trained deep learning models. Similarly, Ahsan et al. [35] proposed the collection and use of image data through a modified VGG16 deep learning model for detecting monkeypox disease. Given the difficulty of identifying monkeypox due to its mild symptoms, Devi et al. [36] suggested an efficient transfer learning method, leveraging the MSLD dataset from the Kaggle repository for easy virus diagnosis. Various other methods, such as the neurofuzzy-based model proposed by Tom et al. [37], have been suggested for detecting variants of the monkeypox virus within the broader pox family.

In the context of healthcare, deep learning has been instrumental in advancing diagnostic accuracy in medical imaging. The CNN model, in particular, has been successfully applied to tasks such as detecting lung abnormalities in chest X-rays [38], identifying brain tumors in MRI scans [39], and classifying skin lesions, including monkeypox, chickenpox, and measles [40]. Studies by Onakpojeruo et al. [41] and Ozsahin et al. [42] have demonstrated that CNN-based architectures are highly effective for brain tumor classification, particularly when combined with generative adversarial network (GAN) models.

Recent studies have also proposed using CNN and other deep learning architectures to enhance the accuracy of ultrasonic-based diagnostics by compensating for data variations. Wang et al. [43] developed a hybrid deep learning model that combines wavelet transforms and transfer learning to enhance the temporal analysis of ultrasonic waveforms, enabling real-time condition monitoring of concrete structures. These studies underscore that deep learning models, especially those incorporating ensemble architectures, can achieve high precision and recall, even with limited or imbalanced datasets.

However, despite their successes, these models often face limitations when dealing with imbalanced datasets, noisy data, and insufficient training samples. To address these challenges, ensemble learning methods, which combine multiple models to improve prediction accuracy, have been explored as viable solutions. To fill this gap, this study introduces an ensemble learning approach that combines Support Vector Machines (SVMs) and transformer models to classify monkeypox lesions. The originality of this method lies in integrating traditional machine learning models (SVMs) with cutting-edge deep learning architectures (transformers), an area that has not been extensively studied in the context of monkeypox lesion classification.

## 3. Dataset Description

The dataset used in this study comprises monkeypox skin lesions collected from publicly accessible sources, including case reports, and websites. A meticulous manual search was conducted to ensure comprehensive data acquisition. During the dataset curation process, multiple strategies were employed to identify and address duplicate images. These included both exact copies and visually similar images from the same patient, identified through manual review. Once duplicates were identified, they were removed to prevent bias in the model, verifying that each image used for training and testing represented a unique instance of a skin lesion. This rigorous approach enhances the generalizability and reliability of the model. The primary objective of this study is to accurately differentiate monkeypox cases from other similar skin lesions, particularly those resembling chickenpox. To facilitate multi-class classification, the dataset was expanded to include images of skin lesions typical of measles, chickenpox, monkeypox, and a “Normal” class representing healthy skin.

Table 1 presents the distribution of the Monkeypox Skin Lesion Dataset (MSLD) in terms of the number of unique patients, class labels, and the number of original images. The dataset includes four distinct class labels: Normal, Measles, Chickenpox, and Monkeypox. The Monkeypox class contains 279 original images of skin lesions associated with monkeypox infections, while the Chickenpox class comprises 107 images of chickenpox lesions. The Measles class includes 91 images depicting measles-related lesions, and the Normal class contains 293 images of healthy skin without lesions. To ensure comprehensive analysis, the MSLD was curated to include images from 55 unique patients across all class labels, ensuring diverse representation and minimizing potential bias from specific individuals. In total, the dataset consists of 770 original images sourced from 162 unique patients.

## 4. Methodology

**Figure 1:**
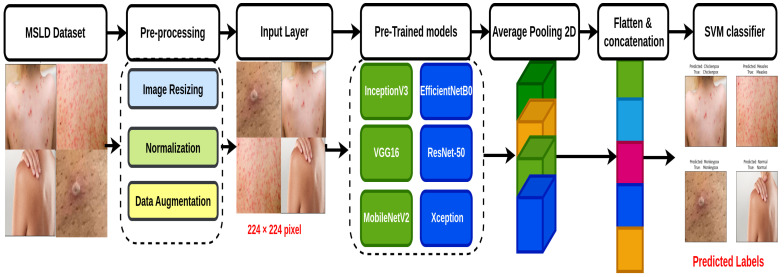
The fundamental steps in the proposed TMS framework.

The proposed TMS model for monkeypox classification is presented in this section. It combines a Support Vector Machine (SVM) with pre-trained CNN architectures including ResNet-50, EfficientNetB0, MobileNetV2, and Xception. Figure 1 illustrates the essential steps of the TMS model. The implementation of the proposed model is divided into three phases: the pre-processing step (Section 4.1), pre-trained models (Section 4.2), and SVM classification (Section 4.3).

### 4.1. The Pre-Processing Step

The pre-processing step is essential for optimizing model performance, particularly when working with medical images that often exhibit significant variability in quality, resolution, and background noise. The detailed pre-processing steps employed in this study are as follows:

1. Image Resizing: The CNN architectures used in this work (such as EfficientNetB0, ResNet50, MobileNetV2, and Xception) require a fixed input size, all images in the dataset were resized to match the input dimensions expected by these models. Specifically, each image was resized to 224 × 224 pixels, which is a standard input size for the selected CNN models. Resizing ensured uniformity and allowed the model to process images efficiently without losing critical visual information.
2. Normalization: To ensure that pixel values were on a consistent scale, we normalized all images by scaling the pixel intensity values to [0, 1]. This was done by dividing the pixel values (originally ranging from 0 to 255) by 255. Image normalization is essential for preventing numerical instability during training, as it ensures that all input data operates within the same range, allowing the deep learning model to converge more effectively.
3. Data Augmentation: To increase the diversity of the training data and reduce overfitting, data augmentation techniques were applied, particularly to classes with fewer samples (e.g., Chickenpox, Measles). The following random augmentations were used during training: (1) Random horizontal and vertical flips: This helped the model learn orientation-invariant features. (2) Random rotations (up to 20 degrees): Rotating images helped the model generalize better to different viewing angles. (3) Zooming (up to 10% in or out): This simulated images captured at varying distances, increasing robustness. (4) Shifts (up to 10% in horizontal and vertical directions): This helped the model become invariant to small translations of the image content.

### 4.2. Pre-Trained Models

This section provides comprehensive details on several pre-trained CNN algorithms, including InceptionV3, EfficientNetB0, ResNet-50, VGG16, MobileNetV2, and Xception. These models have been selected for their exceptional classification performance, and validated across various domains of computer vision, including climate image, and medical image analysis through transfer learning.

Artificial Neural Networks (ANNs) are consists of an input layer, multiple hidden layers, and one output layer. Visually, ANNs can be represented as a network of interconnected nodes and links, where nodes represent variables and links represent weighted parameters. The flow of information through the network is depicted by directional arrows. Unlike traditional three-layer Artificial Neural Networks (ANNs), DL model contains numerous hidden layers with multiple units. When processing data, each frame’s input vector is fed into the ANN’s input units. Subsequently, the output $y_{j}$ of each hidden node *j* is calculated based on inputs from the preceding layer, as follows:

(1) $$y_{j}=\frac{1}{1+e^{m_{j}}},m_{j}=b_{j}+\sum_{i}y_{i}w_{ij}$$

where $w_{ij}$ represents the weight of the connection between nodes *j* and *i*. Each node *j* is assigned a bias $b_{j}$, and the input of node *i* from the preceding layer is denoted as $y_{i}$. Once the activation functions of the nodes in this layer have been calculated, they are transmitted as inputs to the subsequent layer. The output node *j* utilizes the “softmax” function to transform the inputs from the preceding layer into a numerical value representing the classification probability $p_{j}$.

(2) $$p_{j}=\frac{e^{m_{j}}}{\sum_{k}e^{m_{k}}},m_{j}=b_{j}+\sum_{i}y_{i}w_{ij}$$

Convolutional Neural Networks (CNNs) are deep learning architectures designed for processing image data, consisting of alternating convolutional and pooling layers that automatically learn hierarchical feature representations. The convolutional layers apply learnable filters to detect features such as edges, textures, and complex patterns, while the pooling layers reduce spatial dimensions, preserving important information for computational efficiency and robustness to minor input variations. CNNs rely on three key principles: local connectivity, where filters focus on small regions of the input; parameter sharing, which reduces the number of trainable parameters by reusing filters across the image; and translation invariance, enabling the network to recognize features regardless of their location in the image. These principles make CNNs highly effective for tasks like object detection, image classification, and semantic segmentation, maintaining high performance with reduced computational cost.

The VGG16 model is one of the first CNN models developed to enhance performance in large-scale image recognition applications by increasing the model’s depth [44]. It uses 3 × 3 convolutional filters across several layers to prevent the model from overfitting the training data. Additionally, factorized convolution facilitates feature extraction, enabling the model to capture important features. The ResNet architecture was developed based on the concept of residual learning, which leverages residual modules generated after convolution operations and includes Batch Normalization and ReLU non-linearity. These modules optimize the capacity to rapidly forward propagate inputs and extract features with more efficiency. Inception architecture, through its “stacked” Inception modules, demonstrates superior back-propagation despite having fewer parameters than VGG16. Since its initial release, the architecture has undergone several modifications, leading to improved versions being introduced.

Harjoseputro created MobileNet, which serves as the initial model in this study. This model uses the depthwise separable convolution technique to extract features instead of the traditional convolution approach. This technique significantly reduces the number of parameters required for feature extraction, achieving a reduction of 8 to 9 times compared to conventional convolution methods. The model has undergone numerous improvements to enhance its speed and efficiency, including a reduction in the size of feature maps through the use of 1 × 1 convolutions.

In this work, the EfficientNetB0 model is used as an alternative architecture for convolutional neural networks (CNNs). The model was developed by the Google Brain group to enhance CNN accuracy. The model considers the interplay of depth, width, and resolution when designing the architecture. Specifically, the authors emphasize that the term “depth” refers to adding more layers to the existing convolutional model, which has been a common approach to improve model performance. The EfficientNet model also considers the “width” of the network, which refers to the number of channels in each layer, and “resolution”, which is the input image size. By balancing these three characteristics, the EfficientNet model aims to achieve a better trade-off between model performance and the computational resources required.

The ResNet-50 architecture is a CNN that effectively handles the issue of degradation through the utilization of residual learning. This study trained the data without transfer learning using ResNet-50. This decision was made because the initial weights were obtained from a different dataset, making it difficult for them to yield any advantageous outcomes. The essential components of the ResNet model are residual learning and identity mapping, with the skip connection serving as an identity mapping for the block’s input. In traditional convolutional networks, increasing network depth can negatively impact performance due to vanishing gradients, a phenomenon caused by consecutive multiplications that reduce the gradient value during backpropagation. ResNet’s residual blocks ensure that backpropagation is not affected by this issue.

The Xception model, a deep learning architecture proposed by Google Research in 2016, represents an extension of the Inception architecture. Xception leverages depthwise separable convolutions to enhance network efficiency and accuracy. The architecture replaces traditional convolutional layers with a series of depthwise separable convolutional layers, significantly reducing the number of parameters required for training while maintaining the network’s ability to learn complex features. Xception has demonstrated superior performance compared to other leading models on various image classification tasks, leading to its widespread adoption within the computer vision community.

InceptionResNetV2 is a deep learning model that Google announced in 2016 as an extension of the Inception model. The model combines the benefits of residual connections with the Inception architecture, resulting in improved accuracy and reduced computational complexity. InceptionResNetV2 utilizes residual connections to address the vanishing gradient problem, which can occur when training very deep neural networks. The architecture includes multiple Inception modules, each consisting of parallel convolutional layers with different kernel sizes, and a pooling layer. These modules are then connected to residual blocks, which include skip connections that enable faster training and improved accuracy. InceptionResNetV2 has demonstrated superior performance on a range of computer vision tasks and is widely used in both research and industry.

The NASNet-Large is a CNN architecture developed using the neural architecture search (NAS) methodology. The architecture consists of a stack of normal and reduction cells, which are arranged in a specific way to create a network with high accuracy and computational efficiency. The normal cells are used to capture features at a low spatial resolution, while the reduction cells are used to capture features at a higher spatial resolution. The network uses a combination of skip connections, global average pooling, and dropout regularization to improve performance. The NASNet-Large architecture has achieved top performances on various image classification tasks, including the ImageNet dataset.

### 4.3. SVM Classification

The proposed TSM model is based on the Support Vector Machine (SVM), which is well-known for its high generalization abilities and accurate classification capabilities. The SVM utilizes a binary classification technique that is particularly effective in high-dimensional spaces and seeks to identify a hyperplane that maximizes the separation between classes. The SVM model maximizes the distance between two parallel hyperplanes to optimize the separating hyperplane. By producing a well-defined separation, this optimization procedure reduces the classifier’s generalization error across the hyperplane.

Formally, let *D* represent the learning dataset, defined as $D=\{\left(x_{i},y_{i}\right)|x_{i}\in R^{d},y_{i}\in \left\{-1,+1\right\}\}$, where $x_{i}$ denotes the observation features and represents the SVM labels. The learning approach incorporates margin constraints, allowing for instances that may violate these constraints. The slack parameters quantify potential margin violations and introduce penalty criteria to mitigate their impact. The linear training of the SVM is executed through the utilization of the following function:

(3) f(x)=sign(w∗x+b)

The SVM model utilizes a weight vector *w* to represent the orientation of the hyperplane, while *x* denotes an input sample. The bias term *b* serves as a threshold value, determining the hyperplane’s position. An SVM trainer optimizes the hyperplanes for the given data to maximize the margin, which is defined as the shortest distance between the closest data points and the hyperplane, also known as support vectors. This optimization process can be mathematically expressed as follows:

(4) $$\gamma =\frac{2}{\left||\omega |\right|}$$

The SVM algorithm’s classification decisions are based on relevance scores calculated between classes. These scores are assigned equal values, leading to a high score for all labels. The mapping generated through the dot product and kernel trick does not influence the learning time. The SVM classifier exhibits robustness against the curse of dimensionality, particularly in high-dimensional feature spaces. Numerous studies have demonstrated its acceptable performance across diverse domains. Furthermore, the SVM’s ability to refine regression optimization through testing and learning processes is a notable advantage. By incorporating duality properties, margin constraints, and kernel types, the SVM approach can be adapted to address domain-specific challenges, such as nonlinearity and local minima. Moreover, SVMs are capable of effectively distinguishing between different labels and achieving appropriate separation.

### 4.4. The Proposed Framework

Figure 1 shows the essential stages of the proposed TMS model. The initial stage involves pre-processing a dataset comprising 770 images representing four classes: monkeypox, chickenpox, measles, and normal. The outputs generated at this stage are consistent with those obtained using pre-trained CNN architectures in the preceding phase. The input image is passed through pre-trained CNN architectures (EfficientNetB0, ResNet50, MobileNetV2, and Xception), as well as transformer models, which are used for their ability to capture long-range dependencies and contextual information in the images. Each model outputs a vector of features that represent the image in a high-dimensional feature space. The CNNs focus on local and hierarchical features, while transformers capture more global contextual patterns. Once features are extracted from each model (CNNs and transformers), these feature vectors are concatenated into a single unified feature vector.

The second step utilizes seven widely recognized CNN architectures, namely VGG16, MobileNetV2, Xception, ResNet50, InceptionResNetV2, EfficientNetB0, and NASNetLarge, as represented in Figure 1. The pre-trained CNNs (EfficientNetB0, ResNet-50, MobileNetV2, etc.) are used as feature extractors. They are trained on a large dataset of images, and their final layers (before the classification layer) are able to extract essential features. Usually, these features are high-dimensional vectors that represent the image’s content. The extracted features from all architectures are concatenated to create a comprehensive feature representation.

In the last stage, the extracted features from the MobileNetV2, EfficientNetB0, and ResNet50 architectures are aggregated into a single feature vector. This step combines the strengths of both CNN-based local feature extraction and transformer-based global feature extraction, creating a comprehensive representation of the image. The concatenated feature vector is then fed into the Support Vector Machine (SVM) classifier. The SVM is trained to find the optimal hyperplane that separates the different classes (monkeypox, chickenpox, measles, and normal) in this high-dimensional feature space. The choice of SVM is motivated by its ability to perform well with small datasets and effectively handle high-dimensional feature spaces.

## 5. Results Analysis and Discussions

The effectiveness of the TMS model is evaluated in this section using the Google Colab platform. Python is a high-level, flexible programming language that has become the most popular tool for machine learning and scientific computing applications. Python is a powerful, all-purpose, and widely used programming language. It includes a huge variety of libraries and frameworks, especially for deep learning applications, as well as powerful data processing capabilities. For numerous applications, including data preprocessing, forecasting, clustering, classification, visualization, etc., it has all the standard Python packages. Multiple platforms for operating systems support the development and execution of Python programs. Python can be used on a variety of operating systems, including Windows, Mac OS X, Linux, and Solaris. All essential Python libraries were created and released under the General Public License for use in Google Colab, a free cloud. The virtual machine used for the experiments has the following specifications: a 64-bit Linux operating system, an Intel(R) Core i7-9750H CPU running at 2.60 GHz, and 16 GB of memory.

The dataset used in this study, which contains images of Monkeypox, Chickenpox, Measles, and Normal skin lesions, exhibited an imbalance in the distribution of images across these classes. This imbalance posed a potential challenge for the model’s performance, particularly in terms of its ability to generalize well across underrepresented classes. To overcome the effects of class imbalance, we performed data augmentation on the underrepresented classes (Chickenpox and Measles). Data augmentation techniques, such as random rotations, flips, zooms, and shifts, were applied to artificially increase the size of these classes by generating new, slightly altered versions of the original images. This process helped to balance the dataset by providing the model with a more equal number of training samples for each class, without introducing synthetic data that diverged from the original characteristics of the lesions.

The performance of the TMS model was evaluated using a confusion matrix. In addition to accuracy, which can be misleading in imbalanced datasets, precision, recall, and the F1-score were utilized as key evaluation metrics. These metrics provide a more balanced assessment of the model’s performance across all classes, particularly for the minority ones. In this study, both the dataset and the model outputs were meticulously analyzed. Furthermore, experimental evaluations of the proposed model are presented in a step-by-step manner, supported by detailed figures and tables.

### 5.1. Evaluation Metrics

The performance metrics are calculated during the evaluation of the TMS model. The proposed approach was assessed using several metrics, including accuracy, precision, recall, and F1. In binary classification problems, the two output classes are categorized as positive and negative. According to [45], four distinct outcomes can result: True Positive (TP), False Positive (FP), True Negative (TN), and False Negative (FN), as illustrated in Table 2. The classification metrics such as accuracy, precision, recall, and F1-score are computed using Equations (5)–(8), respectively.

Accuracy is the proportion of correctly classified instances out of the total number of instances.

(5) $$Accuracy=\frac{TP+TN}{TP+TN+FP+FN}$$

Recall, also referred to as sensitivity, measures the ratio of correctly predicted positive instances to the total actual positive instances.

(6) $$Recall=\frac{TP}{TP+FN}$$

Precision represents the ratio of correctly predicted positive instances to the total instances predicted as positive.

(7) $$Precision=\frac{TP}{TP+FP}$$

The F1-score provides a balanced evaluation metric by calculating the harmonic mean of precision and recall. It ranges between 0 and 1, where 1 signifies perfect performance, and 0 indicates the poorest performance. The F1-score is computed using the following formula:

(8) $$F1=2\times \frac{Precision\times Recall}{Precision+Recall}$$

The area under the curve (AUC) is a valuable metric ranging from 0 to 1. An AUC of 1 signifies perfect classification with no overlap between classes, whereas an AUC of 0 occurs when all classes are misclassified as others, representing the worst-case scenario.

### 5.2. Experimental Results

**Figure 2:**
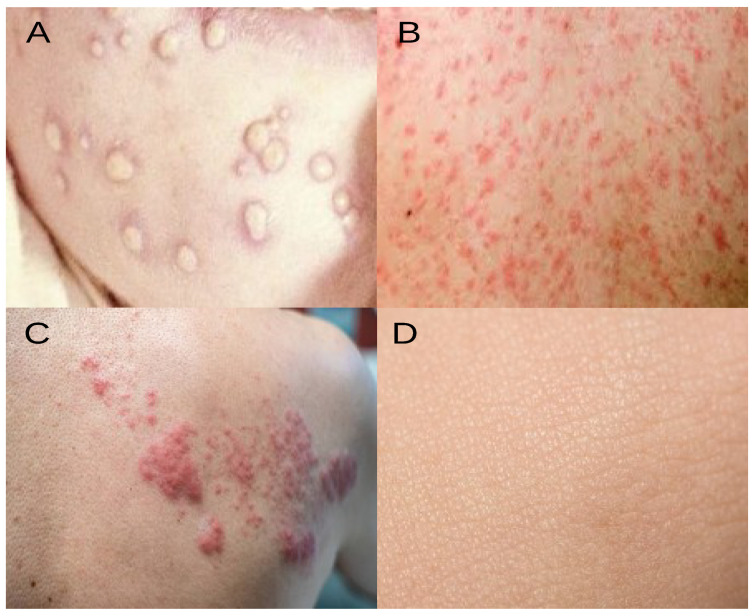
A sample of images from each class utilized in the dataset. These classes include (**A**) Monkeypox, (**B**) Measles, (**C**) Chickenpox, and (**D**) Normal.

The dataset comprises four classes: measles, monkeypox, chickenpox, and normal. Table 1 outlines the label distribution for each class. This study’s primary objective is to use the proposed TMS framework to accurately and effectively predict the correct label for each image in this dataset. Figure 2 illustrates sample images from each class, highlighting representations of monkeypox, chickenpox, measles, and normal. The dataset includes 102 images for Monkeypox, 91 images for Measles, 107 images for Chickenpox, and 293 images for the Normal class.

#### 5.2.1. The Results of CNN Models

The initial stage of implementation involved assessing the performance of seven pre-trained CNN models. For this purpose, the dataset was divided into two subsets: 80% for training and 20% for testing. This division enabled a comprehensive evaluation of the CNN architectures. The training subset was utilized to train the models and update their parameters based on the input data. The testing subset, on the other hand, was used to evaluate the models’ performance on previously unseen data, providing insight into their generalization capabilities. This evaluation process was crucial for determining the models’ effectiveness and reliability in accurately classifying and predicting outcomes using the dataset.

Table 3 shows the performance of the VGG16 model in terms of classification metrics, including accuracy, recall, F1-score, and support for all classes. The model was tested on 21 instances of Chickenpox, achieving a precision of 0.71, indicating its ability to correctly identify positive instances of this class. The recall for Chickenpox was 0.57, demonstrating a moderate capacity for detecting true positive instances. The F1-score for Chickenpox was 0.63, reflecting the harmonic mean of precision and recall. For the Measles class, the VGG16 model was evaluated on 18 images, where it achieved a precision of 0.53, recall of 0.44, and an F1-score of 0.48.

The model was tested on 56 instances of Monkeypox, achieving a precision of 0.72, indicating that it can accurately detect positive instances in this class. The recall for Monkeypox was 0.77, showing the model’s strong ability to identify a large number of true positive instances. The F1-score for Monkeypox was 0.74, reflecting the harmonic mean of precision and recall. The model was also evaluated on 59 instances of Normal, where it performed excellently with a precision of 0.85, demonstrating its ability to correctly identify positive instances in this class. The recall for Normal was 0.90, highlighting the model’s effectiveness in detecting a high proportion of true positive instances. The F1-score for the Normal class was 0.88.

The VGG16 model achieved an overall accuracy of 0.75 across all classes. The macro average precision, recall, and F1-score were calculated as 0.70, 0.67, and 0.68, respectively, considering all classes equally. In contrast, the weighted average precision, recall, and F1-score were 0.75, 0.75, and 0.75, respectively, reflecting the class distribution in the dataset.

#### 5.2.2. The Results of the Proposed Model

**Figure 3:**
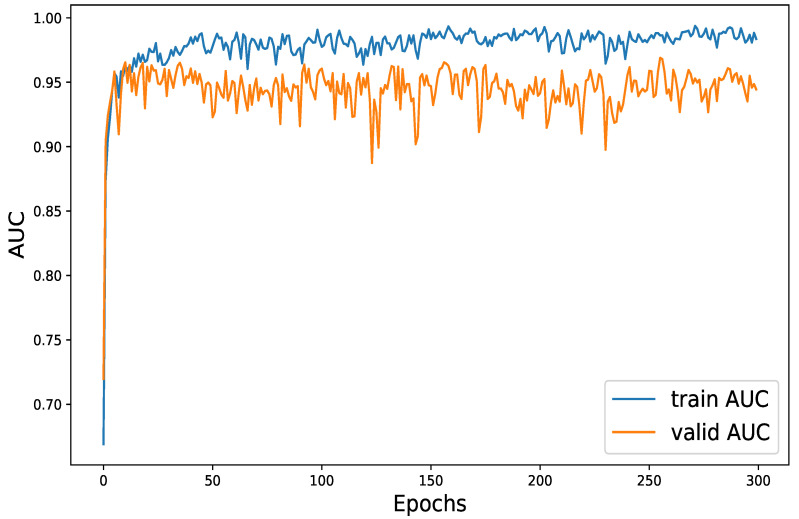
Accuracy curves for training and testing data.

Figure 3 shows the training and validation AUC of the TMS model over 300 epochs. During the first 20 epochs, the training AUC increased rapidly before leveling off. In comparison, the validation AUC also improved, albeit at a slower pace, reaching its peak at about 250 epochs.

**Figure 4:**
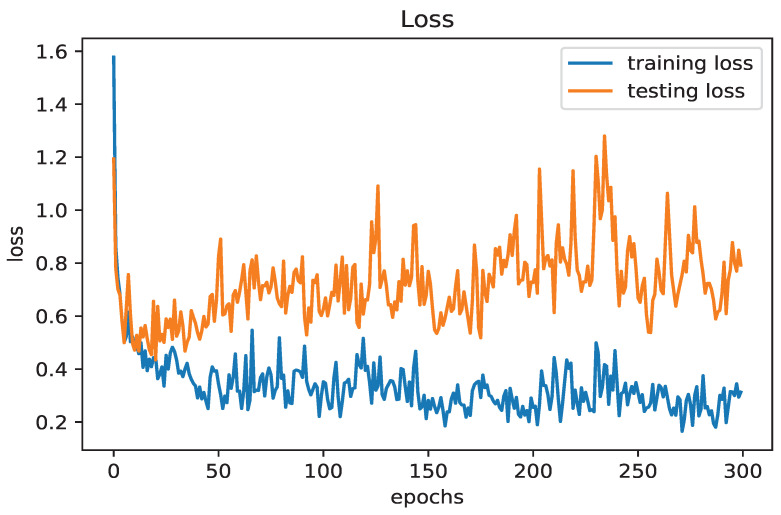
Loss curves for training and testing data.

Figure 4 presents the loss curves for both the training and testing data. The x-axis represents the number of epochs (ranging from 0 to 300), while the y-axis shows the loss values (ranging from 0.2 to 1.6). The blue training loss curve remains relatively stable with minor fluctuations, while the orange testing loss curve shows greater variability and an overall upward trend. The legend indicates that the blue line represents "training loss" and the orange line represents "testing loss". These curves provide insights into the performance of the model during training, highlighting potential issues such as overfitting and insufficient generalization. The increasing testing loss, contrasted with the decreasing training loss, suggests that the model may be struggling to generalize to unseen data, pointing to the need for further adjustments to improve performance and reduce overfitting.

**Figure 5:**
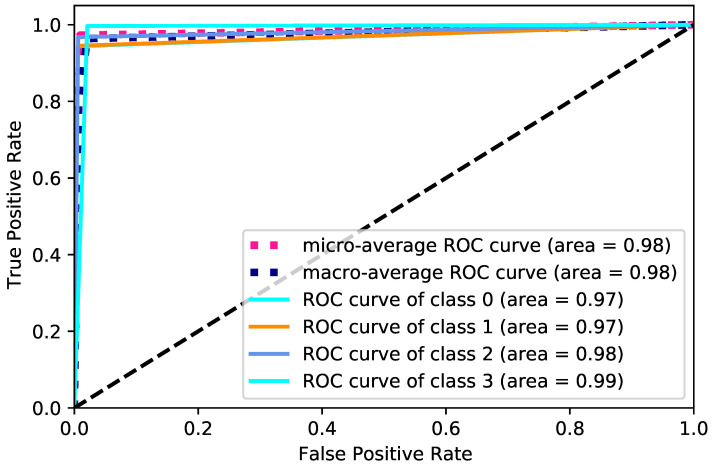
ROC curves for all classes.

Figure 5 shows the receiver operating characteristic (ROC) curves for a multi-class classification task. The x-axis represents the false positive rate, ranging from 0 to 1, while the y-axis represents the true positive rate (sensitivity or recall), also ranging from 0 to 1. The figure includes the micro-average ROC curve, macro-average ROC curve, and the individual ROC curves for each class. The micro-average ROC curve, shown in blue, demonstrates an impressive area under the curve (AUC) of 0.98, reflecting excellent overall performance across all classes. Similarly, the macro-average ROC curve in orange also has a high AUC of 0.98, indicating strong average performance across all individual classes. The individual ROC curves for each class (class 0, class 1, class 2, and class 3) show AUC values between 0.97 and 0.99, indicating good ability to distinguish between positive and negative instances within each class.

**Figure 6:**
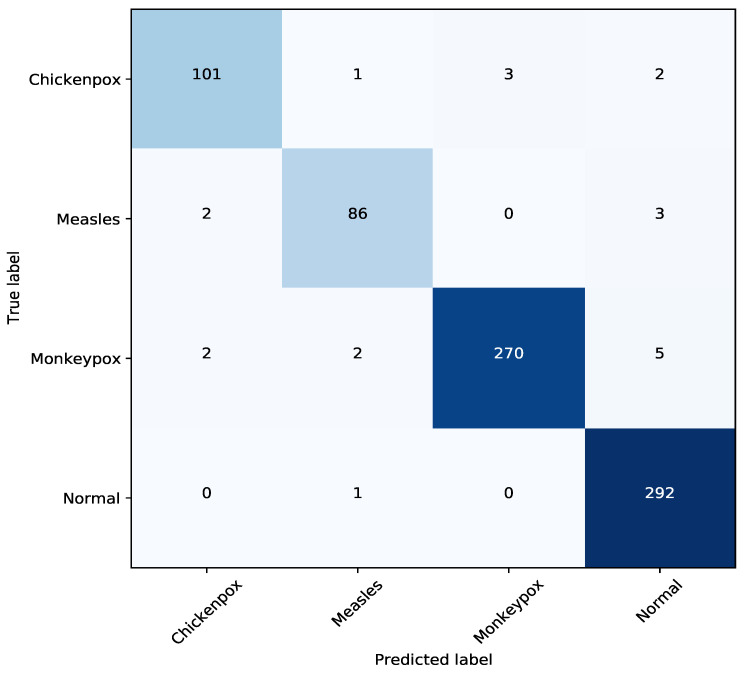
The confusion matrix of the ensemble model.

Figure 6 presents the confusion matrix of the TMS model, showcasing its performance on the classification task. The matrix provides a detailed overview of how the model correctly and incorrectly classified instances of each class. For example, the value “101” in the first row and first column indicates that 101 images were accurately identified as chickenpox. On the other hand, the value “2” in the second row and first column shows that 2 images were misclassified as chickenpox when they actually belonged to the measles class.

The confusion matrix reveals important insights into the model’s strengths and weaknesses. High values along the diagonal suggest that the model correctly classifies a significant portion of instances for each class. The diagonal elements represent correctly classified instances, while the off-diagonal elements highlight misclassifications. By analyzing the misclassification patterns, areas for potential model improvement can be identified.

**Figure 7:**
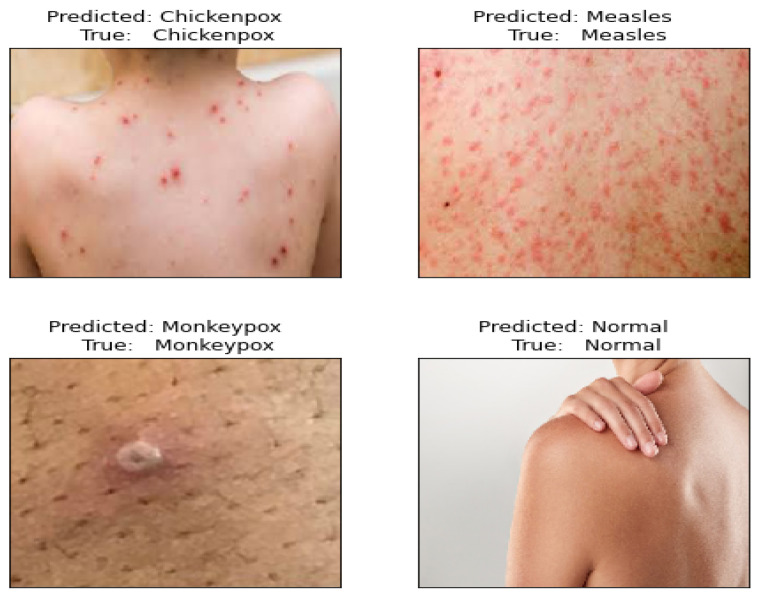
An example of the predicted images using the proposed model.

Figure 7 illustrates the validation results of the proposed model using new images. The model successfully categorized the new images into four distinct classes: chickenpox, measles, monkeypox, and normal. Each class is represented by a separate row in the figure. In each row, the leftmost column displays the original input image, while the subsequent columns show the predicted images for each respective category. The model’s capability to accurately predict the correct class for each image highlights its effectiveness in image classification tasks.

Table 4 compares the TMS model against other pre-trained models based on accuracy, recall, precision, and F1. Among the models assessed, the TMS model outperformed the others, achieving an accuracy of 95.45%. It demonstrated a precision of 95.51%, reflecting its strong ability to accurately identify positive instances. The recall value of 95.45% further highlights its effectiveness in detecting true positive instances. The F1 score, which combines both precision and recall, was 95.46% for the TMS model.

The EfficientNetB0 model achieved an accuracy of 85.45%, with a precision of 80.05%, indicating that it could identify positive instances while minimizing false positives. Its recall value was 85.45%, reflecting its strong performance in recognizing true positive instances. The F1 score for EfficientNetB0 was 85.45%. The ResNet50 model also showed solid performance with an accuracy of 84.03%, precision of 85.05%, and recall of 84.03%. Its F1 score was 84.03%, indicating a balanced trade-off between precision and recall.

The MobileNet model performed well with an accuracy of 90.26%, a precision of 90.24%, and a recall of 90.25%, showcasing its ability to identify positive instances and true positives with minimal error. The F1 score for MobileNet was 90.26%.

Similarly, the MobileNetV2 model achieved an accuracy of 86.75%, with a precision of 86.77% and a recall of 86.75%. Its F1 score was 86.76%. The Xception, NASNetLarge, and InceptionResNetV2 models demonstrated lower performance, with accuracy values of 73.12%, 64.29%, and 59.61%, respectively.

## 6. Discussion

This study highlights the effectiveness of deep learning models in detecting monkeypox skin lesions, with promising classification performance. However, certain limitations impact the broader applicability of the results. The primary drawback is the dataset’s lack of diversity in patient populations, which may affect the model’s generalization ability across different groups. To enhance performance across various factors, it is crucial to include a larger sample size with better representation of geographical, racial, and gender diversity. A more diverse dataset would enable the TMS model to generalize effectively to various patient populations.

Another limitation arises from the use of pre-trained weights from the ImageNet dataset for transfer learning. Since ImageNet does not contain skin lesion images, pre-training the model on a multi-source dermatoscopic image dataset would improve its performance and ability to generalize. Such a dataset would allow the model to learn features specific to skin lesions, thereby enhancing its detection capabilities.

Additionally, the dataset used in this study, primarily obtained via web scraping, lacks essential metadata, including information on the disease’s onset, the patient’s clinical history, and the disease stage—critical factors for accurate diagnosis. Including this metadata would provide valuable context, improving both the reliability and clinical applicability of the models. To address these gaps, international collaboration is needed to compile a larger, more representative dataset that incorporates comprehensive metadata.

The current dataset’s web-scraped nature introduces further challenges, such as the absence of vital clinical parameters like patient history, disease progression, and staging. These are crucial for a thorough diagnostic assessment. Building a more robust diagnostic framework requires collaborative international efforts to establish a standardized dataset with diverse demographic representation and detailed clinical metadata. Such a dataset would significantly enhance the clinical relevance and practical utility of automated diagnostic systems.

Future research could aim to overcome the current limitations by expanding the dataset to include a broader range of patient populations, ensuring better geographical, racial, and gender representation. Incorporating relevant metadata such as patient history, disease stage, and progression would further enhance the accuracy and applicability of the models. Additionally, investigating alternative pre-training strategies, such as using specialized dermatoscopic image datasets, could improve the model’s ability to detect skin lesions, leading to greater accuracy and generalization.

The findings from this study demonstrate the significant potential of artificial intelligence in detecting monkeypox skin lesions. By including real images collected from various regions, future classification tasks could more accurately represent real-world conditions. The best results were obtained by combining features extracted from pre-trained models like MobileNetV2, ResNet50, and EfficientNetB0 with an SVM classifier. This work demonstrates the importance of using advanced pre-trained models and SVM classifier to improve detection capabilities in skin lesion classification tasks.

## 7. Conclusions

This study presents the Monkeypox Skin Lesion Dataset (MSLD), an open-source dataset designed for the classification of monkeypox skin lesions. The TMS model, which combines pre-trained CNN architectures, including EfficientNetB0, ResNet-50, MobileNetV2, and Xception, with a support vector machine (SVM), was used in this study to classify monkeypox. The proposed ensemble model achieved a classification accuracy of 95.45%. The effectiveness and robustness of the model were further confirmed through comprehensive performance metrics and visual representations of the results. Experimental findings demonstrate the superiority of the TMS model compared to existing methodologies in the task of monkeypox classification. To improve diagnostic accuracy, future directions for this research involve assessing the proposed method using large-scale datasets and conducting engineering optimization problems to comprehensively evaluate its strengths and weaknesses. Future research may also extend the framework to include multimodal data integration, such as clinical metadata and patient history. Additionally, investigating the model’s generalizability across diverse demographic groups and varying imaging conditions would strengthen its clinical applicability. Implementing explainable AI techniques could also provide insights into the model’s decision-making process.

## Figures and Tables

**Table 1:** The class label distribution for the MSLD dataset.

Class Label	# Original Images	# Unique Patients
Monkeypox	279	55
Normal	293	107
Chickenpox	107	55
Measles	91	55
Total	770	162

**Table 2:** The parameters of the confusion matrix.

	Predicted	Predicted
Actual	TP	FP
Actual	FN	TN

**Table 3:** The results of the classification report for VGG16 model.

Class	Precision	Recall	F1-Score	Support ^1^
Chickenpox	0.71	0.57	0.63	21
Measles	0.53	0.44	0.48	18
Monkeypox	0.72	0.77	0.74	56
Normal	0.85	0.90	0.88	59
Accuracy			0.75	154
Macro avg	0.70	0.67	0.68	154
Weighted avg	0.75	0.75	0.75	154

^1^ Support refers to the number of instances for each label in the test set.

**Table 4:** Performance comparison between the TMS model and the transformer models.

Model	Accuracy	Precision	Recall	F1
TMS	95.45	95.51	95.45	95.46
MobileNet	90.26	90.24	90.25	90.26
MobileNetV2	86.75	86.77	86.75	86.76
EfficientNetB0	85.45	80.05	85.45	85.45
ResNet50	84.03	85.05	84.03	84.03
Xception	73.12	70.48	73.12	73.12
NASNetLarge	64.29	50.26	64.29	64.29
InceptionResNetV2	59.61	53.24	59.61	59.61

## Data Availability

The dataset is available on the Kaggle website: https://www.kaggle.com/datasets/dipuiucse/monkeypoxskinimagedataset (accessed on 10 September 2024).
